# An unusual type I ribosome-inactivating protein from *Agrostemma githago* L.

**DOI:** 10.1038/s41598-020-72282-2

**Published:** 2020-09-21

**Authors:** Christoph Weise, Achim Schrot, Leonie T. D. Wuerger, Jacob Adolf, Roger Gilabert-Oriol, Simko Sama, Matthias F. Melzig, Alexander Weng

**Affiliations:** 1 Institute of Chemistry and Biochemistry, Freie Universität Berlin, Thielallee 63, 14195 Berlin, Germany; 2 Institute of Pharmacy, Freie Universität Berlin, Königin-Luise-Str. 2+4, 14195 Berlin, Germany; 3 Tentamus Analytics GmbH, An der Industriebahn 5, 13088 Berlin, Germany; 4 Department of Experimental Therapeutics, British Columbia Cancer Research Centre, Vancouver, BC V5Z 1L3 Canada

**Keywords:** Plant sciences, Secondary metabolism, Biochemistry, Enzymes, Proteins

## Abstract

*Agrostemma githago* L. (corn cockle) is an herbaceous plant mainly growing in Europe. The seeds of the corn cockle are toxic and poisonings were widespread in the past by consuming contaminated flour. The toxic principle of *Agrostemma* seeds was attributed to triterpenoid secondary metabolites. Indeed, this is in part true. However *Agrostemma githago* L. is also a producer of ribosome-inactivating proteins (RIPs). RIPs are *N*-glycosylases that inactivate the ribosomal RNA, a process leading to an irreversible inhibition of protein synthesis and subsequent cell death. A widely known RIP is ricin from *Ricinus communis* L., which was used as a bioweapon in the past. In this study we isolated agrostin, a 27 kDa RIP from the seeds of *Agrostemma githago* L., and determined its full sequence. The toxicity of native agrostin was investigated by impedance-based live cell imaging. By RNAseq we identified 7 additional RIPs (agrostins) in the transcriptome of the corn cockle. Agrostin was recombinantly expressed in *E. coli* and characterized by MALDI-TOF–MS and adenine releasing assay. This study provides for the first time a comprehensive analysis of ribosome-inactivating proteins in the corn cockle and complements the current knowledge about the toxic principles of the plant.

## Introduction

*Agrostemma githago* L. (corn cockle) is an annual herbaceous waist-height plant from the carnation family (Caryophyllaceae). It blooms in splendid pink-purple flowers—the name *Agrostemma* means “garland of the fields”.

In former times *Agrostemma githago* L. predominantly grew on corn fields as a troublesome weed. In the course of grain harvest, *Agrostemma* seeds were inadvertently processed to flour. According to reports from the nineteenth century lethal poisonings in humans occurred following the consumption of contaminated flour^1^. The Russian military commissariat allowed the consumption of maximally 5 g *Agrostemma* seeds per kg bread for a soldier. This was a dosage, which could already cause severe poisonings^1^.

With the use of weed killers in the twentieth century and the development of improved seed cleaning techniques the corn cockle gradually disappeared from the fields. Nowadays *Agrostemma githago* L. is virtually extinct in the wild in many parts of Europe and is considered as an endangered species.

The seeds of *Agrostemma githago* L. are known to contain triterpene saponins^2^ with gypsogenin as aglycon. The toxicity of the seeds is usually attributed to the triterpene saponin content in the seeds. It is known that saponins at higher concentrations are able to lyse eukaryotic cells^3–5^. In addition to triterpene saponins the seeds of *Agrostemma githago* L. contain type I ribosome-inactivating proteins (type I RIPs). These RIPs are N-glycosylases (EC 3.2.2.22) that remove an essential adenine (A^4324^) from the 28S ribosomal RNA. This leads to an irreversible inhibition of protein synthesis and subsequent cell death^6^.

Type I RIPs consist only of the N-glycosylase domain, whereas type II RIPs such as ricin from *Ricinus communis* L. additionally contain a lectin domain which binds with high affinity to galactose molecules on the cell surface. The physiological role of type I RIPs in the plant cell is not completely understood, however they have been reported to provide protection against herbivores^7,8^ and viruses^9^.

The family of the Caryophyllaceae contains a considerable number of species that biosynthesize type I RIPs. Prominent examples are saporin from *Saponaria officinalis* L. or dianthin from *Dianthus caryophyllus* L^10^.

In 1983 Stirpe et al*.* isolated three type I RIPs from *Agrostemma githago* L., which were termed agrostin 2 (29.2 kDa, IP = 7.7), agrostin 5 (25.5 kDa, IP = 8.7) and agrostin 6 (27 kDa, IP = 8.75)^11^. According to their mechanism of action type I ribosome-inactivating proteins contribute significantly to the toxicity of *Agrostemma githago* L.

In 2003 Hebestreit et al. showed that triterpene saponins from the seeds of *Agrostemma githago* L., increased the cytotoxicity of agrostin in a synergistic manner^12^. The reason for this increase lies in the fact that triterpene saponins enhance the endosomal escape process of the type I ribosome-inactivating proteins within the cell^13^. The endosomal escape is thus a prerequisite for RIP-related toxicity. This synergistic toxicity of agrostin with triterpene saponins contributes significantly to the toxicity of the seed material.

Recently we have identified *Gypsophila elegans* M. Bieb (Caryophyllaceae) as yet another plant able to co-synthesize triterpene saponins and type I RIPs in seeds^14^.

Astonishingly, although a commercial product called “agrostin from *Agrostemma githago* seeds” (Sigma A7928) has been available for several years (its distribution was discontinued around 2005), never any molecular data pertaining to agrostin has been published. This is in contrast to other type I RIPs where such data was reported at an early stage: The amino-acid composition for two RIPs from *Saponaria officinalis* L. and a RIP from the latex of the sandbox tree *Hura crepitans* (Euphorbiaceae) was published in the very same paper in which the purification of the three forms of agrostin was originally reported^11^. The N-terminal sequence of saporin-6, a RIP from *Saponaria officinalis* L., was available as early as 1985^15^.

In order to fill this lack of knowledge we aimed to isolate, characterize and identify type I RIPs from *Agrostemma githago* L.

## Results and discussion

### Isolation of agrostin from seeds of *Agrostemma githago* L

**Figure 1:**
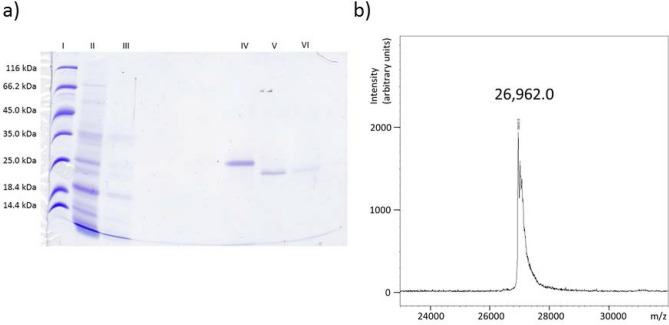
(**a**) SDS-PAGE (12%) of Agrostin_seed, isolated from the seeds of *Agrostemma githago* L. by immuno-affinity chromatography I: Marker; II: Extract (diluted 1:10, PBS); III: Wash fraction after application of the extract; IV: Agrostin_sigma; V: first fraction of the eluted agrostin; VI second fraction of eluted agrostin. (**b**) Intact protein mass as determined by MALDI-TOF MS analysis.

Agrostin was isolated by affinity chromatography using an anti-agrostin antibody raised against commercially available agrostin. Using this approach allowed for a direct one-step purification from the aqueous extract from *Agrostemma* seeds by which agrostin was obtained in high purity, as shown in Fig. 1a. In comparison with the commercial agrostin from Sigma-Aldrich a small mass shift was observed in the SDS-gel. This might be due to a glycosylation of agrostin from Sigma-Aldrich. Glycosylation of agrostin had been reported previously^1^.

Mass-spectrometric analysis of the intact protein yielded a main peak at 26,962 ± 7 Da, with two side peaks at a slightly higher total mass. Whether these are due to artificial or physiological protein modifications or represent very similar protein isoforms could not be established. The isolated agrostin henceforward is referred to as Agrostin_seed and agrostin obtained from Sigma-Aldrich as Agrostin_sigma.

### MALDI-TOF–MS

**Figure 2:**
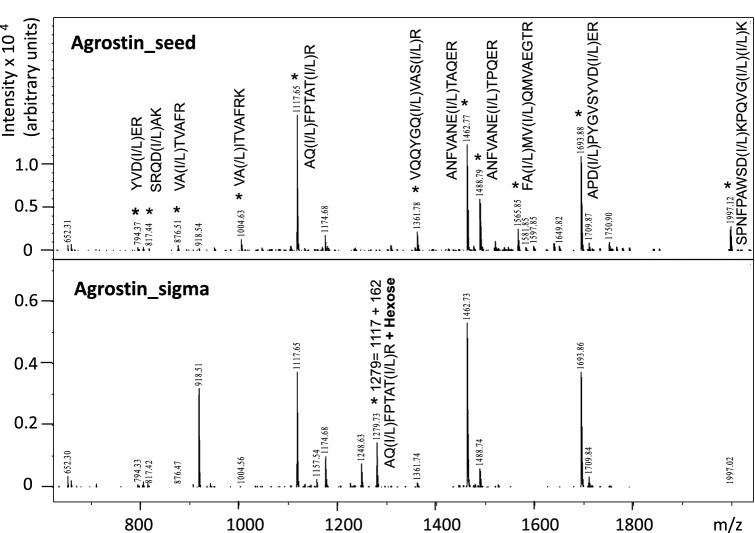
Peptide mass fingerprint (trypsin in-gel digestion) of Agrostin_seed (top) and the commercial protein Agrostin_sigma (bottom). The peptides marked by an asterisk were sequenced by MALDI-TOF–MS: [M + H] 794.37; 817.44; 876.51; 1,004.63; 1,117.65; 1,361.78; 1,462.77; 1,488.79; 1565.85; 1693.88; 1997.12 and the underlying peptide sequences are indicated for the each mass. The additional peptide at M + H = 1,279 in Agrostin_sigma was identified as an O-glycosylated form of the peptide AQLFPTATIR (M + H = 1,117) carrying one single hexose residue (∆m = 162).

The isolated Agrostin_seed was further subjected to in-gel digestion using trypsin and AspN protease (data not shown) and the resulting peptides were analysed by MALDI-TOF–MS (Fig. 2). A number of selected peptides (depicted with an asterisk in Fig. 2) were fragmented and their sequences were determined de novo from the MS/MS spectra. Assuming from its total mass that agrostin consists of approximately 245 amino acids, these peptides represent roughly 40% of its total sequence. A comparison with the tryptic peptide map of Agrostin_sigma (Fig. 2) showed that the two proteins are essentially identical. Interestingly, one additional peptide in the Sigma protein at M + H = 1,279 was identified as an O-glycosylated form of the peptide AQLFPTATIR (M + H = 1,117, Pos. 095-104) carrying one single hexose residue. The precise O-glycosylation site was not identified. Supporting the value of this observation, however, the bioinformatic prediction using the NetOGlyc server over the whole length of the agrostin sequence yields the highest O-glycosylation probability for the two threonine residues T100 and T102 contained in precisely this peptide. A higher degree of glycosylation—there might be more glycosylation events that remained undetected—might explain the slightly different migration behaviour in SDS-PAGE seen in Fig. 1.

For the determination of the full sequence and to identify further RIPs in the transcriptome of *Agrostemma githago* L. RNAseq was performed. Prior to RNAseq an expression analysis of agrostin in different developmental stages of *Agrostemma githago* L. was conducted.

### Expression analysis of agrostin in *Agrostemma githago* L

**Figure 3:**
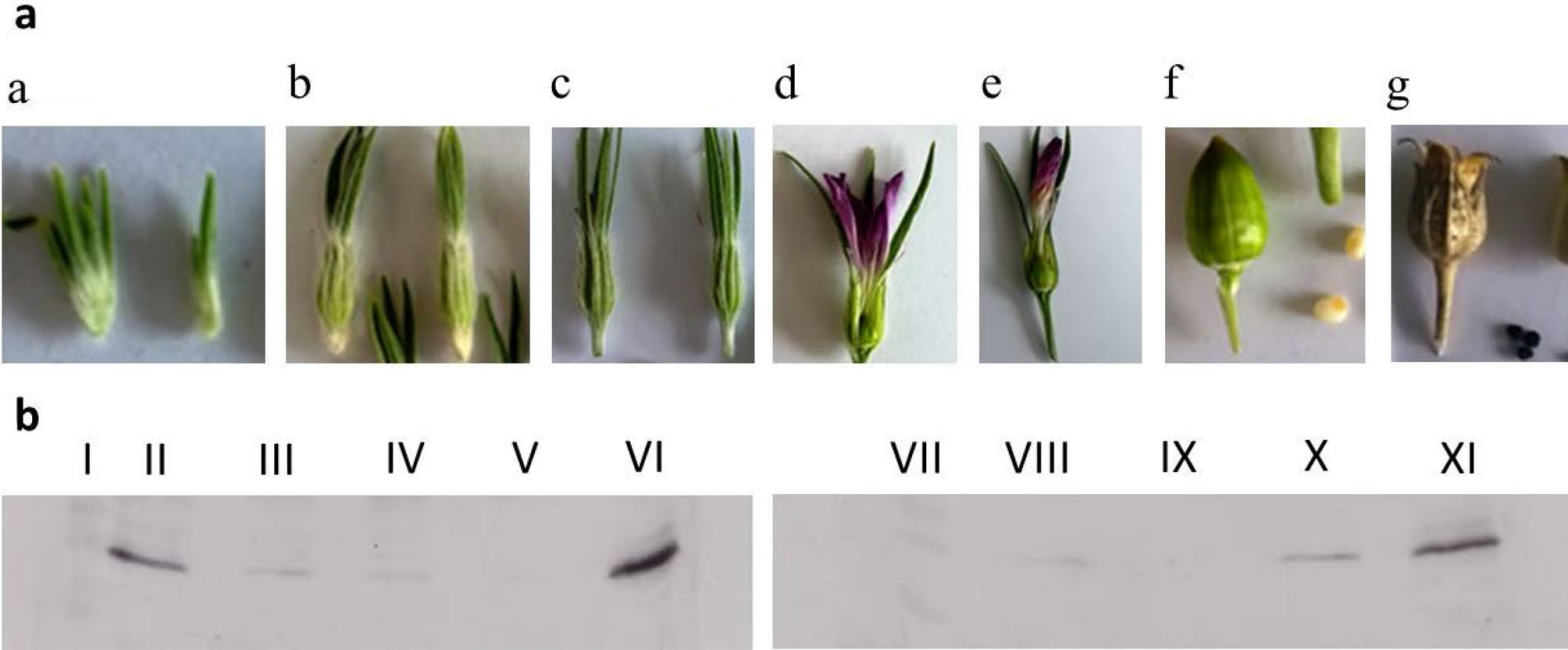
(**a**) Different development stages of *Agrostemma githago* L. a: Appearance of the sepals; b: Appearance of the petals; c: Appearance of the seed capsule; d: Petals fully developed and colored; e: Petals parched, growing seed and seed capsule; f: Maturation of seed and seed capsule, seeds white-yellow colored; g: Loss of sepals, seeds black colored and fully developed, seed capsule open. (**b**) Western blot analysis of the extracts from stages a-g using the anti-agrostin antibody. I: Marker; II: Stage a; III: Stage b; IV: Stage c; V: Stage d; VI: Agrostin_seed; VII: Marker; VIII: Stage e; IX: Stage f; X: Stage g; XI: Agrostin_seed.

For the RNA extraction (RNAseq) we aimed to identify those developmental stages in which *Agrostemma githago* L. shows a high expression of agrostin. For this reason *Agrostemma githago* L. was seeded and grown to different developmental stages (stage a–g, Fig. 3a). The extracts of the plant material derived from the different developmental stages were analysed by western blot using the anti-agrostin antibody. As shown in Fig. 3b agrostin is already expressed in young plants (stage a) but it could also be detected in stage g. The expression of agrostin apparently fluctuates during plant development.

For the extraction of RNA (RNAseq) the plant material from stage a was used.

### Determination of amino-acid sequences

**Figure 4:**
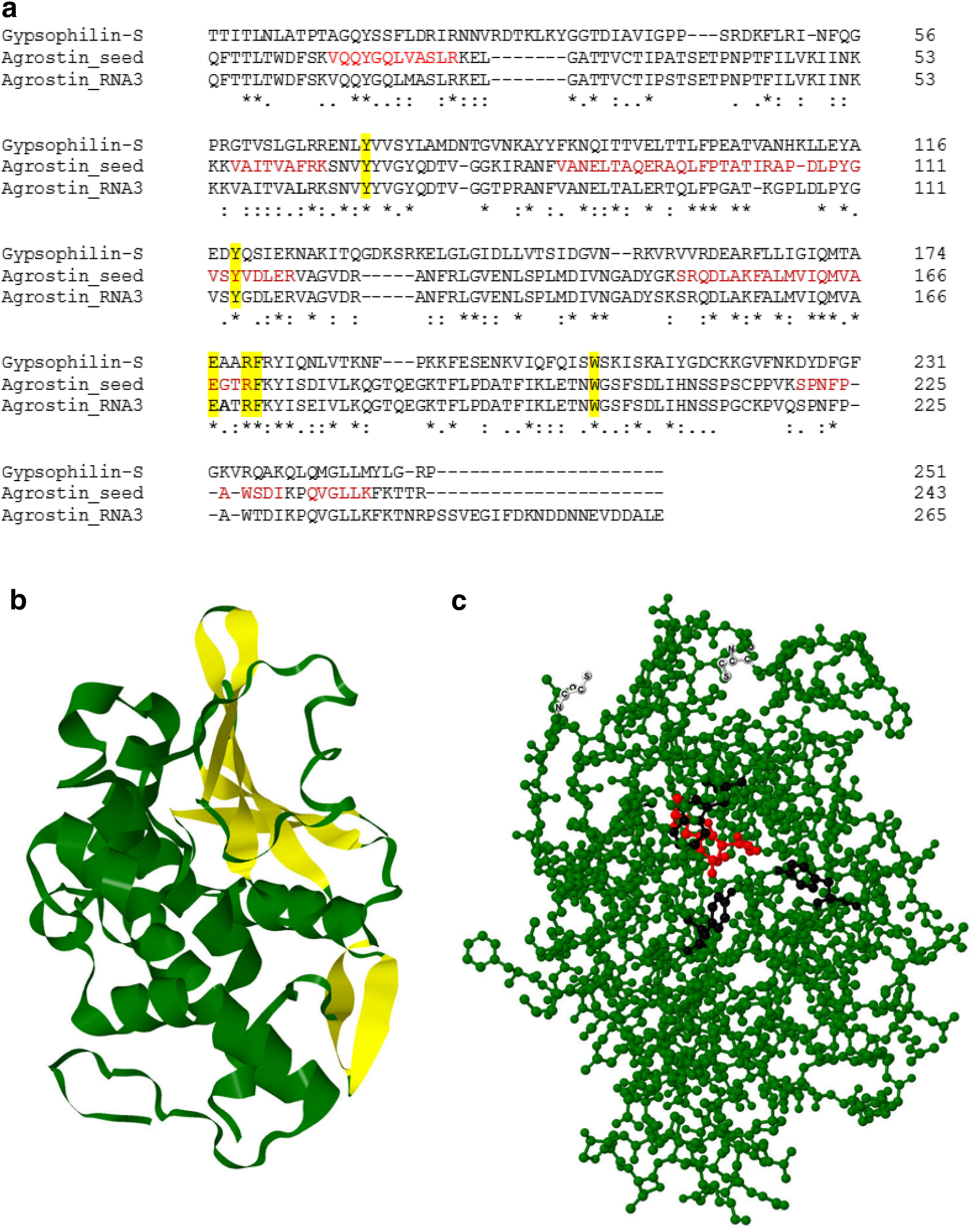
(**a**) Alignment of gysophilin-S, a type I RIP from *Gypsophila elegans* M.Bieb, Agrostin_seed, isolated from the seeds and agrostin (Agrostin_RNA3) from the early development stage a (Fig. 3a) of *Agrostemma githago* L. Functionally relevant conserved amino acids are highlighted in yellow. Peptides sequenced by MS/MS analysis, covering the sequence of the Agrostin_seed are shown in red. Aligment was performed using the Clustal Omega multiple sequence alignment tool^16^. (**b**) Hypothetical tertiary structure of Agrostin_seed using Phyre2^17^ and Jmol^24^. The N-terminal region is rich of β-sheets highlighted in yellow, whereas the C-terminal region is dominated by α-helices. (**c**) Ball-and-stick model of Agrostin_seed. The amino acids Glu 167 and Arg 170, representing the active site, are shown in red, Tyr 68, Tyr 114 and Trp 202, representing the substrate binding site are shown in black. Cys 32 and Cys 216, a potential conjugation site to other biomolecules, are depicted in white.

By transcriptome analysis we identified the sequence Agrostin_RNA3 shown in Fig. 4a, which is very similar to the peptide sequences shown above. However, we found a substantial number of discrepancies between this sequence and the peptide sequences obtained by MALDI-TOF MS-analysis, e.g. while a peptide with the sequence VAITVA**F**RK (M = 1,003.62) was identified by MS/MS-analysis, the corresponding sequence in Agrostin_RNA3 was VAITVA**L**RK with a clearly different mass (M = 969.63). Similar small differences existed for most of the analysed peptides. We therefore concluded that the Agrostin_RNA3 sequence represents an agrostin isoform, which is present in stage a (Fig. 3a) of the development, but not exactly the protein purified from the seeds.

By combining the results obtained on the peptide level by mass spectrometry and on the level of the nucleotide sequences by transcriptome sequencing, we succeeded in assembling the sequence of Agrostin_seed corresponding to the protein isolated from the seeds (Fig. 4a).

Agrostin_RNA3 and Agrostin_seed are very similar (92% sequence identity), representing agrostin isoforms.

For Agrostin_seed ambiguities remain in three positions between the protein data and the RNA seq data (V019M, N042S, S228T). In these cases we used the amino acids according to the peptide sequences for the final sequence presented in Fig. 4, since we assume that they represent more direct evidence for the protein purified from the plant. The discrepancies might be due to sequencing inaccuracies or might arise from the diversity of the biological material used in this study. Intriguingly we found one peptide in two versions (R.ANFVANELT**A**QER, M = 1,461.7, and R.ANFVANELT**P**QER, M = 1,487.7) pointing to a certain degree of heterogeneity within the Agrostin_seed fraction.

Agrostin_seed shows the typical features of a type I RIP such as gypsophilin-S from *Gypsophila elegans* M.Bieb^14^. Its theoretical molecular mass calculated from the sequence is 26,966.0 Da (M + H, average) which is in good agreement (Δ = 148 ppm) with the experimental value (Fig. 1b). The theoretical isoelectric point as calculated with the tool ProtParam is 9.43, which is somewhat higher than the experimental values given by Stirpe for the different agrostin peaks observed in his work (7.7 for peak 2, 8.7 for peak 5 and 8.75 for peak 6)^11^.

The hypothetical three-dimensional structure of agrostin generated using Phyre2^17^ shows the typical composition of other type I RIPs, which consists of an N-terminal β-sheet-rich domain followed by an α-helix-rich succession (Fig. 4b).

Using the Agrostin_seed amino-acid sequence, a similarity search using the protein basic logic alignment tool BLASTp yielded the highest percentage identity value of 36% to the type I RIP bouganin from *Bougainvillea spectabilis* Willd. This is surprising since *Bougainvillea spectabilis* Willd belongs to another plant family (Nyctaginaceae). The value of 36% is remarkably low; sequence identity is even lower with other RIPs from plants from the same plant family (Caryophyllaceae), 30% with gypsophilin-S, 27% with saporin-6, and 26% with dianthin. This finding is even more striking as the similarity between these three proteins is much higher (in the range of 80% sequence identity).

It highlights the exceptional position that Agrostin_seed adopts among the type I RIPs from the carnation family.

Type I RIPs, especially saporin, are used for the construction of targeted anti-tumor toxins, consisting of monoclonal antibodies and type I RIPs as toxin portions^18^. Clinical studies have also been performed with such kind of conjugates^19,20^ and a huge number of saporin-based antibody conjugates, addressing different targets, are commercially available^21^. In this context agrostin is a new interesting option for generating conjugates with potentially lower immunogenicity. Immunogenicity of the RIP portion is a big problem and differs quite a lot among RIPs^22^. The conjugation of toxins to monoclonal antibodies is achieved by chemical linkers. Due to their intrinsic nucleophilicity thiols (cysteines) are well suited for chemical conjugations via disulfide formation or coupling via maleimides^23^. However, in order to take advantage of thiol coupling chemistry the cysteines must be accessible on the surface of the protein. Agrostin_seed contains the cysteines Cys 32 and Cys 216. Molecular analysis using Jmol^24^ shows that the thiol of Cys 32 might be accessible for chemical modification, whereas that of Cys 216 is rather oriented towards the core of the protein (Fig. 4c). This offers the possibility of a site-specific modification with chemical linkers such as maleimide cross-linkers and coupling to monoclonal antibodies with defined coupling stoichiometry.

### Hypothetical RIP-sequences from the transcriptome of *Agrostemma githago* L

The analyses of the RNAseq data set revealed 7 different RIP sequences. It is likely that the translation of these transcripts depends on factors such as development, infections or abiotic stress^25^. The derived protein sequences were aligned using CLUSTALW^16^ and signal sequences were determined by SignalP 5.0^26^ (The alignment is depicted in the supplementary information, Fig. S1) and the functionally relevant amino acids are present throughout all sequences. There are only very few plants with such a variety of RIPs in their transcriptomes. Table 1 shows the results of the alignments against Agrostin_seed. Except for Agrostin_RNA3 all other Agrostin_RNA sequences show rather low percentage identity values. By performing a BLASTp search bouganin from *Bougainvillea spectabilis* Willd. was found as best match for most of the agrostin RNA sequences. This is striking, since *Bougainvillea spectabilis* Willd. belongs to a different plant family (Nyctaginaceae) and there is obviously no similarity to other RIPs from more closely related plants of the same family of the Caryophyllaceae. However phylogenetic analysis showed an evolutionary relationship of type I RIPs within the Caryophyllales^27^ and a considerable number of type I RIPs from the Caryophyllaceae such as petroglaucin from *Petrocoptis glaucifolia* (Lag.) or pyramidatin from *Vaccaria hispanica* (Mill.) Rauschert are still not sequenced^28,29^.

**Table 1:** Alignment using BLASTp^30,31^ of sequences obtained from transcriptome sequencing against Agrostin_seed and BLASTp database.

Protein name	Query coverage (%)	Sequence identity (%)	E-value	IP	Length (amino acid)	BLASTp (NCBI*)
Agrostin_seed	100	100	0	9.4	243	Chain A, rRNA N-glycosylase from *Bougainvillea spectabilis* (36%)
Agrostin_RNA1	89	35	1e−44	9.4	300	Bouganin (36%)
Agrostin_RNA2	85	28	5e−19	9.7	273	RIP from *Beta vulgaris* (33%)
Agrostin_RNA3	91	91	2e−169	6.8	265	Bouganin (36%)
Agrostin_RNA4	95	35	1e−46	9.4	300	Bouganin (35%)
Agrostin_RNA5	83	27	1e−16	8.8	287	RIP from *Atriplex patens* (35%)
Agrostin_RNA6	90	37	1e−44	9.2	296	Bouganin (36%)
Agrostin_RNA7	75	53	7e−82	6.9	312	RIP from *Bougainvillea spectabilis* (40%)

The isoelectric point (IP) was determined using the ExPASy ProtParam tool^32^.

*Database: All non-redundant GenBank CDS translations + PDB + SwissProt + PIR + PRF excluding environmental samples from WGS projects.

### Cytotoxic activity of agrostin

The cytotoxicity of Agrostin_seed was investigated in ECV-304 cells by impedance-based real-time analysis. In previous studies we have shown that particular triterpene saponins augment the cytotoxicity of type I RIPs by improving the endosomal escape of internalized type I RIPs^13^.

Following endocytosis into the cell, type I RIPs need to escape from lysosomes into the cytosol. This is a very important step in the course of the toxin routing, since the target organelles (ribosomes) are located in the cytosol.

**Figure 5:**
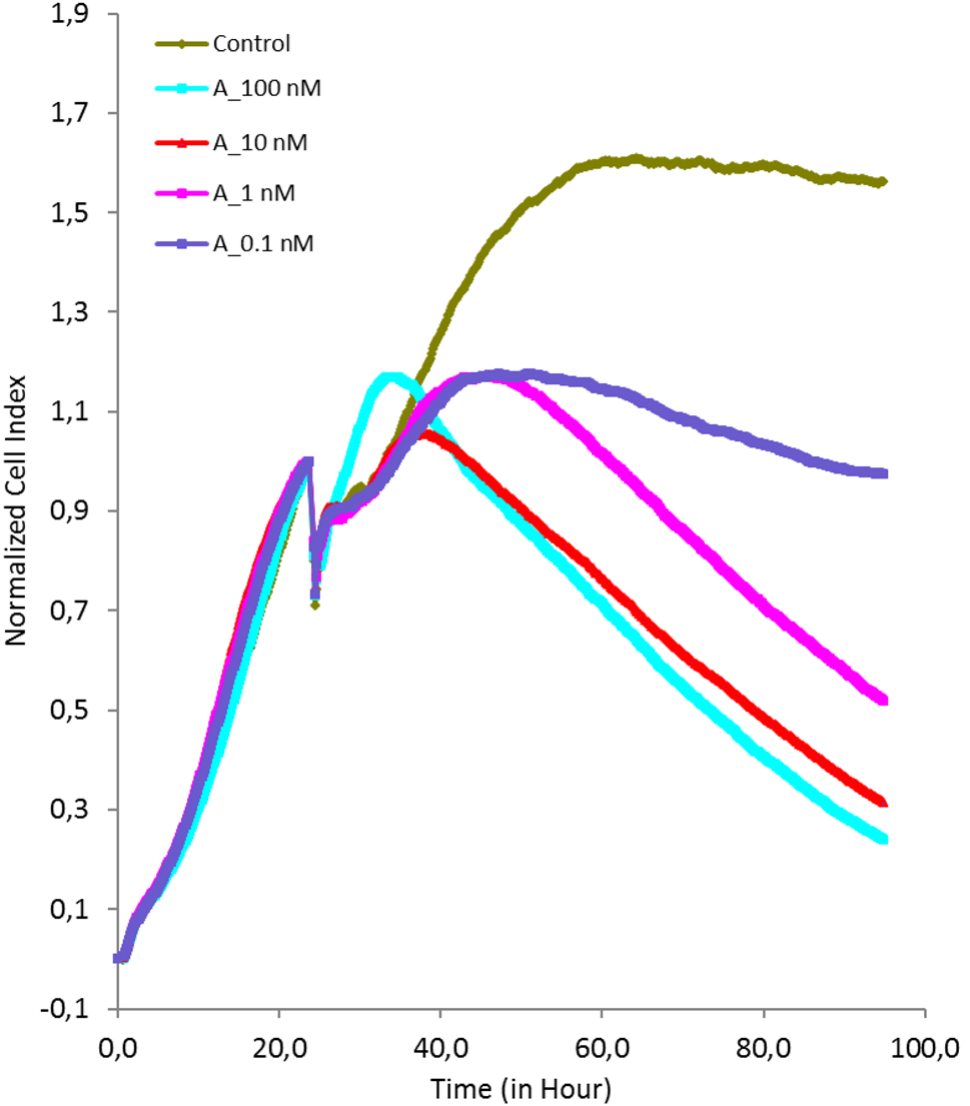
Impedance-based live cell imaging of ECV-304 cells. After an incubation period of 24 h Agrostin_seed (A) was added at different concentrations (0.1–100 nM) with SO1861, which is a triterpene saponin isolated from *Saponaria officinalis* L. Cells were continuously monitored for 96 h. SO1861 enhanced the cytotoxicity of Agrostin_seed by improving the delivery of the protein to the ribosomes.

For this reason we combined agrostin with the a non-toxic concentration of the triterpene saponin SO1861^33^ (Fig. 5).

### Recombinant expression of Agrostin_seed

**Figure 6:**
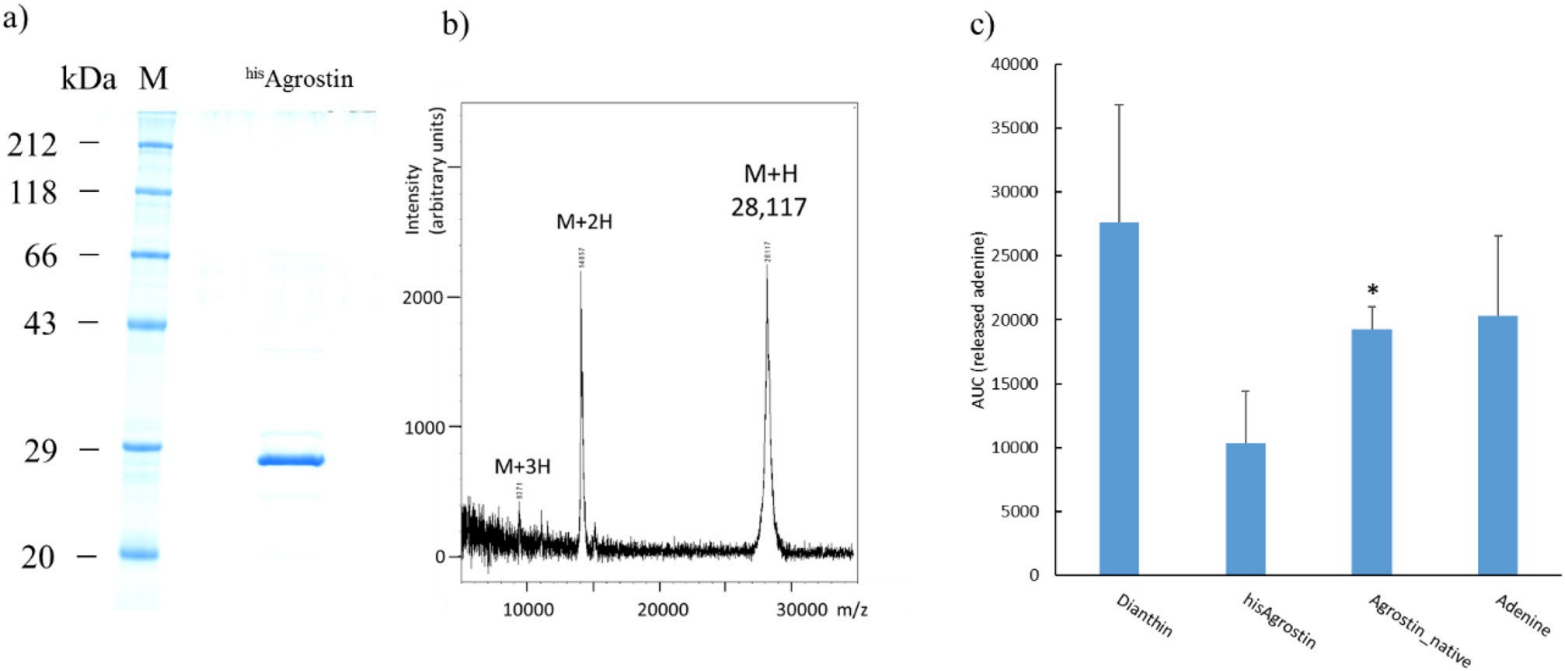
Recombinant expression of ^his^Agrostin in *E. coli*. (**a**) SDS-PAGE of ^his^Agrostin, Coomassie Brilliant Blue stain. ^his^Agrostin appeared at ~ 29 kDa. (**b**) MALDI-TOF–MS spectrum of intact ^his^Agrostin. The mass of ^his^Agrostin was determined as 28,117 Da. (**c**) TLC-based adenine releasing assay of ^his^Agrostin and native Agrostin. ^his^Agrostin showed N-glycosylase activity against an oligo (A) substrate. However native Agrostin (1 µg), that was isolated from the seeds (Agrostin_seed) exhibited significantly higher activity compared to ^his^Agrostin (1 µg). Recombinant dianthin (0.25 µg) was used as positive control for a type I RIP and single adenine (0.25 µg) was used as chromatographic control. *significant to ^his^Agrostin, *t* test, *p* ≤ 0.05.

Based on the amino-acid sequence of Agrostin_seed, a codon-optimized nucleic acid sequence including the sequence for an N-terminal 8 × His affinity tag was generated by gene synthesis. The recombinant Agrostin_seed is henceforward referred to as ^his^Agrostin. ^his^Agrostin was expressed in *E. coli*. and following the isolation by metal affinity chromatography one prominent band at around 29 kDa could be seen on the SDS-PAGE (Fig. 6a). The exact mass of ^his^Agrostin was determined by MALDI-TOF–MS as 28,117 Da. (Fig. 6b). This value is in very good agreement with the theoretical mass calculated from the sequence (28,119 Da). The identity of ^his^Agrostin was further verified by its peptide mass fingerprint (data not shown) and MALDI ISD sequencing (see supplementary information, Fig. S2).

The enzymatic activity of ^his^Agrostin was determined in a densitometric TLC assay^34^, which is based on the RIP-catalysed release of adenine molecules from an artificial substrate.

As shown in Fig. 6c ^his^Agrostin showed enzymatic activity, even though its activity was not as high as the activity of native Agrostin_seed. This could be due to a partially uncorrect folding of ^his^Agrostin during expression in *E. coli*. In future studies this issue might be solved by optimzing the expression conditions in *E. coli*. However, the recombinant type I RIP dianthin from *Dianthus caryophyllus* L., which was used as positive control, showed an even a higher activity. This could be also due to a higher substrate specifity of ^his^Agrostin and native Agrostin compared to dianthin, DNA not being the natural substrate of RIPs.

## Methods

### Seed material

Seeds (Agrostemmae semen, AGRO 26/80) from *Agrostemma githago* L. were obtained from the *Bundesanstalt für Züchtungsforschung und Kulturpflanzen* (BAZ) in Gatersleben, Germany. Seeds (200 g) were grinded and defatted by Soxhlet extraction using petroleum ether overnight. The material was air-dried and extracted at 4 °C by 500 ml PBS supplemented with protease inhibitor (cOmplete Protease Inhibitor Cocktail, Roche, Mannheim, Germany). After 12 h the extract was centrifuged at 6,000 g for 20 min and then subjected to ultracentrifugation (Optima L-90 K, Beckmann Coulter GmbH; 30,000 rpm, 30 min, 4 °C). The clear supernatant was subjected to affinity chromatography (see below).

### Isolation of agrostin

For the isolation of Agrostin_seed an anti-agrostin antibody was generated in rabbits (Pineda antibody service, Berlin, Germany). For the immunization, commercial agrostin (Sigma-Aldrich, Steinheim, Germany) was used. Following ammonium sulfate precipitation of the serum the IgG fraction was isolated by protein A-based column chromatography (Pierce Protein A Agarose, Thermo Fisher Scientific). The antibodies were eluted by 0.1 M glycine, pH 2.5, 4 °C and neutralized by Tris buffer (1 M, pH 9.0, 4 °C). For the isolation of anti-agrostin antibodies, 100 µg of commercial agrostin was immobilized on NHS-Activated Agarose Spin Columns (Pierce, Thermo Fischer Scientific). After applying the IgG fraction and washing (PBS), anti-agrostin antibodies were eluted by 0.1 M glycine, pH 2.5, 4 °C and neutralized (Tris buffer 1 M, pH 9.0, 4 °C). Fractions were pooled, dialysed against PBS and analysed by SDS-PAGE (12%).

For the isolation of Agrostin_seed, anti-agrostin antibodies were immobilized on NHS-Activated Agarose Spin Columns (Pierce, Thermo Fisher Scientific). The *Agrostemma* seed extract (500 ml) was gradually applied to the column. After washing (5 ml PBS, 4 °C), bound Agrostin_seed was eluted by adding 5 ml 0.1 M glycine, pH 2.5, 4 °C. In total 13 fractions (each 0.5 ml) were collected, neutralized (see above) and dialysed against PBS. Two fractions contained agrostin. Protein concentration was determined by BCA assay and fractions were analysed by SDS-PAGE (12%), Coomassie Brilliant Blue staining.

### Mass spectrometry

Proteins and peptides were analysed by matrix-assisted laser desorption ionization-time of flight mass spectrometry (MALDI-TOF–MS) using an Ultraflex-II TOF/TOF instrument (Bruker Daltonics, Bremen, Germany) equipped with a 200 Hz solid-state Smart beam laser. The mass spectrometer was operated in positive mode. Samples were spotted using the dried-droplet technique. Intact protein mass was determined in linear mode (LP_ProtMix) using sinapinic acid as the matrix (saturated solution in 33% acetonitrile/0.1% trifluoroacetic acid) and spectra were acquired over an m/z range of 3,000–40,000. The mass accuracy obtained in linear mode measurements in the higher mass range (> 10 kDa) was estimated as ± 1 ‰.

Peptides generated by in-gel trypsin digestion (modified from Shevchenko et al.^35^) were measured in reflector mode (RP_PepMix) using α-cyano-4-hydroxycinnamic acid (saturated solution in 33% acetonitrile/0.1% trifluoroacetic acid) as the matrix and spectra were typically acquired over an m/z range of 600–4,000. Data was analysed using FlexAnalysis 2.4. software. MS/MS spectra of selected tryptic peptides were acquired in the LIFT mode^36^ and de novo interpretation of the fragment spectra was performed manually. In-source decay (ISD) was used to generate N-terminal c ions and C-terminal (z + 2) ions from the intact purified and acetone-precipitated recombinant protein using 1,5-diaminonaphthalene (1,5-DAN) as matrix. Spectra were recorded in the positive reflector mode (RP_PepMix) in the m/z range 800–4,000. Mass accuracy here was < 100 ppm.

### Agrostin expression in different development stages

In order to identify the right time point for RNA isolation for the transcriptome sequencing different maturation states of growing *Agrostemma githago* L. plants were analysed for agrostin expression. For this purpose 7 development states of the plant were selected: Stadium a: 3 months after seeding, appearance of the sepals, stadium b: appearance of the petals, stadium c: appearance of the seed capsule, stadium d: Petals fully developed and colored, stadium e: Petals parched, growing seed and seed capsule, stadium f: Maturation of seed and seed capsule, seeds white-yellow, stadium g: Loss of sepals, seeds black and fully developed, seed capsule open.

The fresh plant material was snap-frozen in liquid nitrogen, grinded and defatted. The material was extracted by PBS (cOmplete Protease Inhibitor Cocktail; Roche, Mannheim, Germany) at a concentration of 100 mg/ml and analysed by western blot using the anti-agrostin antibody (1:7,500) as primary and a goat anti-rabbit antibody (IgG, H and L Chain Specific Peroxidase Conjugate, Merck, 1:2,000) as secondary antibody. Amersham Hybond ECL, (GE Healthcare Lifesciences), ECL (Enhanced chemoluminiscence)-reagent and an Optimax TR (M&S Laborgeräte, Heidelberg, Germany) were used for development.

### Transcriptome sequencing (RNAseq)

Total RNA was isolated from plants in stadium a (see above). For this purpose, the frozen plant material was grinded in liquid nitrogen. RNA was extracted from 102 mg plant material using TriSure and Direct-zol RNA Miniprep kits (Zymo Research, Freiburg, Germany). Extracted RNA was stored at − 80 °C. The sample was analysed by agarose gel (1%) electrophoresis. Concentration was determined to 2.50 µg/µl (NanoDrop 1,000, Thermo Fischer Scientific). The RNAseq was performed using an Illumina MiSeq V3 (LGC Genomics GmbH, Berlin, Germany).

The raw results were demultiplexed with Illumina’s data analysis software CASAVA and then cleaned of adapter sequences. Forward and reverse reads were combined using^37^ BBMerge 34.48.

The resulting sequences were deconcatemerised and quality trimmed to include only reads with an average P. hred quality score of at least 30. Based on these 12,115,767 reads, a de novo assembly was performed using Newbler v 2.9 in cDNA mode, and putative ORF identification was done by Transdecoder. Trinotate was used to annotate the resulting transcontigs and predicted peptides to identify those sequences with a high similarity to known RIPs.

Besides using Newbler, we performed another assembly with Mira^38^. This assembly was based on all quality trimmed reads with a sequence that could be translated into either of the peptide fragments obtained by MALDI-TOF MS and all other reads similar to these originally filtered reads.

### Impedance-based real-time measurements

The toxicity of the isolated Agrostin_seed was investigated by impedance-based real time imaging. For this purpose ECV-304 cells (ACC 310, Leibniz Institut, DMSZ, Braunschweig, Germany) were seeded in 100 µl (5,000/well) DMEM medium, supplemented with 10% FBS in 96-well E-Plates (xCELLigence RTCA System, ACEA Biosciences)^13,39^. After 24 h, Agrostin_seed was added (final conc. 0.1–100 nM). In order to scrutinize a potential synergistic toxicity with triterpene saponins^13^ SO1861^33^ was added at a final concentration of 1 µg/ml. Cells were continuously imaged for 96 h.

### Recombinant protein expression

The codon-optimized coding sequence was established by gene synthesis (General Biosystems, Inc., Morrisville, USA) and cloned into the expression vector pET11d (Merck, Darmstadt, Germany). The coding sequence contained an N-terminal 8 × His tag for metal affinity chromatography. The construct ^his^Agrostin_pET11d was transformed into competent *Escherichia coli* Rosetta 2 (DE3) cells (Merck, Darmstadt, Germany). The bacterial culture was expanded to 3.2 l using LB medium containing 50 µg/µl ampicillin and incubated until an optical density at 600 nm (OD_600_) between 0.9 and 1.2 was reached. Protein expression was induced using 1 mM isopropyl β-D-1-thiogalactopyranoside (AppliChem, Darmstadt, Germany) for 3 h at 37 °C and 200 rpm. The expression was stopped by centrifugation for 10 min at 5,000 g and 4 °C. Subsequently, the bacterial pellets were resuspended in 20 ml PBS and stored at − 20 °C. The bacterial suspensions were thawed and lysed by sonication (Branson Sonifier 250, G. Heinemann, Schwäbisch Gmünd, Germany). The lysates were centrifuged at 15,800 g and 4 °C for 10 min and imidazole was added to the supernatant to a final concentration of 20 mM. ^his^Agrostin was purified using Ni-nitrilotriacetic acid agarose affinity chromatography (Protino Ni–NTA agarose, Macherey–Nagel, Düren, Germany). The bound protein was eluted using increasing imidazole concentrations (20, 50, 75, 125 and 250 mM, 5 ml for each concentration) and analysed by SDS-PAGE [12% acrylamide (w/v) gel]. The protein was dialysed against 2 l PBS and protein concentration was determined using the bicinchoninic acid assay (Pierce BCA Protein Assay, Thermo Scientific, Waltham, MA, USA).

### N-glycosidase assay

The N-glycosidase activity was determined using an adenine releasing assay with an artificial substrate. The assay is described in detail elsewhere^34^.

Briefly, the substrate consists of the DNA oligonucleotide 5′-A_30_-3′ (A30). Once the N-glycosidic bond is cleaved, released adenine is separated from the reaction mixture by Thin Layer Chromatography (TLC) on silica gel 60 glass plates. The glass plates are then scanned by a TLC-densitometer (TLC Scanner 4, CAMAG, Berlin, Germany) at 260 nm. The RIP-mediated release of adenine is determined by calculating the Area Under the Curve (AUC).

## Supplementary information

Supplementary file1
