# Supplementary material for: An unusual type I ribosome-inactivating protein from Agrostemma githago L
Source: Sci Rep. 2020 Sep 21;10:15377. doi: 10.1038/s41598-020-72282-2 (PMC7506001; doi:10.1038/s41598-020-72282-2)
Supplement: Supplementary file 1 — Supplementary file1 [file 41598_2020_72282_MOESM1_ESM.docx]

**An unusual type I ribosome-inactivating protein from *Agrostemma githago* L.**

Christoph Weise^1^, Achim Schrot^2^, Leonie T.D. Wuerger^2^, Jacob Adolf^3^, Roger Gilabert-Oriol^4^, Simko Sama^2^, Matthias F. Melzig^2^, Alexander Weng^2^*

^1^ Freie Universität Berlin, Institute of Chemistry and Biochemistry, Thielallee 63, 14195 Berlin, Germany

^2^ Freie Universität Berlin, Institute of Pharmacy, Königin-Luise-Str. 2+4, 14195 Berlin, Germany

^3^ Tentamus Analytics GmbH, An der Industriebahn 5, 13088 Berlin, Germany

^4^ Department of Experimental Therapeutics, British Columbia Cancer Research Centre, Vancouver, BC V5Z 1L3, Canada

*Corresponding author, [alexander.weng@fu-berlin.de](mailto:alexander.weng@fu-berlin.de), Tel.: +49 30 838 51265

Fig. S1

Alignment of agrostin sequences from transcriptome sequencing with the sequence of Agrostin_seed. Signal sequences were determined by SignalP 5.0 and removed prior to alignment.

The alignment was performed using CLUSTALW. Relevant amino acids are highlighted in yellow.

Agrostin_RNA_1 ------------------IPSNTTQGTLQYDTVKWPLDLGTAS---------------KP

Agrostin_RNA_4 ------------------IPSNTSRGTLQYDTVRWPLDLGTAS---------------KP

Agrostin_RNA_6 --------------------------TLQYDTVRWKLDLETAS---------------KP

Agrostin_RNA_3 ----------------------------QFTTLTWDFSKVQQ------------------

Agrostin_seed ----------------------------QFTTLTWDFSKVQQ------------------

Agrostin_RNA_7 ------------------KLKAPPPPPPVYPTITWNLNDYET------------------

Agrostin_RNA_2 -----REDNAINSPNLIFNSSTTDPNSLLSNTLPIPINLDLTILAVER-----------A

Agrostin_RNA_5 -----HEDANNNNPNNFYSNSTT-------NILTIDLG-HLTAPAKAKDVGKQISQDRGI

: :.

Agrostin_RNA_1 YTDFLSSLRGKLKAGEVCKFPVTTKTPASNKKFILVDIIETNKPKPRTITLALLASDGYF

Agrostin_RNA_4 YTDFLSSLRAKLKAGEVCKFPVTTKTPASNKKFILVDIIES-KPQPRTITLALLASDGYF

Agrostin_RNA_6 YTDFLSSLRGKLKAGEVCKFPVTTKTPASNKKFILIDIIDT-KPQPRTITLALRASDAYF

Agrostin_RNA_3 YGQLMASLRKELGATTVCTIPSTSETPN--PTFILVKIINK---KKVAITVALRKSNVYY

Agrostin_seed YGQLVASLRKELGATTVCTIPATSETPN--PTFILVKIINK---KKVAITVAFRKSNVYY

Agrostin_RNA_7 YDNLMVSLRTQLGVGKVCNIPVTSQFPTREKLFILVEIINL---KQKIITLAFRQSNVYF

Agrostin_RNA_2 YNDVITRIRKSVQGNHFYHDIPIMVAPSQPTQFIEVRLFATRGRDRISVSLAIRKTDLYV

Agrostin_RNA_5 YNGVIEQIRQSVRGEIYYNGIPMIAQRTRPPQYTVVSLVGLRGDEQIALSIAIQNSDLYT

* .: :* .: : : :. . :::*: :: *

Agrostin_RNA_1 LGFRD-ELNGAPRANFVADNFAEWKASDVFGTTVKDANKKKLPFQSSYSSMESTAKLGGE

Agrostin_RNA_4 LGFRD-ESNGAPRANFVADNFAEWKASDVFGTTVKEANKKKLPFQSSYSSMESTAKLGGE

Agrostin_RNA_6 LGFRD-EFNGVPRANFVEDDFAEWKASDVFNN-VKEANKKKLPFQSSYDSMESAAKLVK-

Agrostin_RNA_3 VGYQD-TVGGTPRANFVANELTALERTQLFPGATKGP--LDLPYGVSYGDLERVA---GV

Agrostin_seed VGYQD-TVGGKIRANFVANELTAQERAQLFPTATIRA--PDLPYGVSYVDLERVA---GV

Agrostin_RNA_7 VGYKDKLTGGASRAFFIKDELTATEKSLLFTDATIR---KDLPFGGSYPDIERVA---GK

Agrostin_RNA_2 VAYAD---PHLRKAFFFSD--FKDIEPSIFP-TRVPFAKSMLSFESGYVPLENTS---GL

Agrostin_RNA_5 VAYAD---PKLHKAFFFKDEKFKDIQASIFPTTDVPFAKVVLPFLSSYPSLEDNA---KT

:.: * :* *. : . :* *.: .* :* :

Agrostin_RNA_1 GRKNLPLGINNLNDLIDQIYGKPYKGVRKSNDEIGVNLAKFGLTVVQMVAEAVRFKYIED

Agrostin_RNA_4 GRKNLPLGINNLNDLIDQIYGKPYKGVRKSNDEIGVNLAKFGLTVVQMVAEAVRFKYIED

Agrostin_RNA_6 GRKGLPIGIQNLDDLIEQIYGKPYKGISKSNEEIGVSLAKFGLTVVQMVAEAVRFKYIED

Agrostin_RNA_3 DRANFRLGVENLSPLMDIVNGADYS---KS----RQDLAKFALMVIQMVAEATRFKYISE

Agrostin_seed DRANFRLGVENLSPLMDIVNGADYG---KS----RQDLAKFALMVIQMVAEGTRFKYISD

Agrostin_RNA_7 SRANINLGVQALSTAIDSIYGLDHA---KI----GKELATFALVAIQMISEATRFTYIER

Agrostin_RNA_2 KRPELQMSLEKLIFSMRAVSGREPQP---------KNQAKFLLYATQAFSEAARFKYLQ-

Agrostin_RNA_5 RRFELEMSLEKLVFSMLPVHGKAKTDG--------KNQAKFLLYAVQAVSESVRFKYVQ-

* : :.:: * : : * . *.* * . * .:*..**.*:.

Agrostin_RNA_1 AVVKGGIG-KKSFKPGDDIISLVKEWGKISEAIH--VSAGNKPACKNIPKIDYPTGVTTV

Agrostin_RNA_4 AVVKGGIGSKKSFKPGDDIISLVKEWGKISEAIH--VSAGNKPACKNIPKIDYPTGVTTV

Agrostin_RNA_6 AVVKEGIGKKKSFKPGDDIISLVKEWGKISQAIH--VSAADKKACEKISGFDYPKGVTTV

Agrostin_RNA_3 IVLKQGTQEGKTFLPDATFIKLETNWGSFSDLIH--NSSPGCKPVQ------SPNFPAWT

Agrostin_seed IVLKQGTQEGKTFLPDATFIKLETNWGSFSDLIH--NSSPSCPPVK------SPNFPAWS

Agrostin_RNA_7 TVKAEGMFG--NFLPNPTFIELETNWGTFSDLIR--NSD-CSKPVT------SPKFPEFK

Agrostin_RNA_2 -KKFGGVVEANTGGVDYLVKALENEWDTISTAVKQAVRRMLKPPVVLAHRNGSRWQVESV

Agrostin_RNA_5 -KKFGGV--ATVFEKDYGVPAVENEWKTISKAVKDAINGKLQPPLVLAHPDGTKREVENV

* . . : .:* .:* :: .

Agrostin_RNA_1 DGLRSWVSLAKFKKGK-----------------------------------VSNTTVDAA

Agrostin_RNA_4 DGLRSYVSLMKFKKGK-----------------------------------VSNTTVDD-

Agrostin_RNA_6 DGIRNYVSLVKFKKSK-----------------------------------VSNTTVDAA

Agrostin_RNA_3 D-IKPQVGLLKFKTN--RPSSVEGIFDKNDD----------------------NNEVDDA

Agrostin_seed D-IKPQVGLLKFKTT--R------------------------------------------

Agrostin_RNA_7 D-IKNEVGLLKFLKANAPPPPKKGIFDNNDDGDKFDKFLKTSPPPLKKGIFDNNDENGDQ

Agrostin_RNA_2 DEIKPNIGILKFVK----------------------------------------------

Agrostin_RNA_5 KDIKPEIGILQYV-----------------------------------------------

. :: :.: ::

Agrostin_RNA_1 AAVMEVAELAFM-----

Agrostin_RNA_4 AAVMEVAELAFM-----

Agrostin_RNA_6 AVVTEVAELAFM-----

Agrostin_RNA_3 LE---------------

Agrostin_seed -----------------

Agrostin_RNA_7 FVRMPFSFCDECFMHEI

Agrostin_RNA_2 -----------------

Agrostin_RNA_5 -----------------

Fig. S2


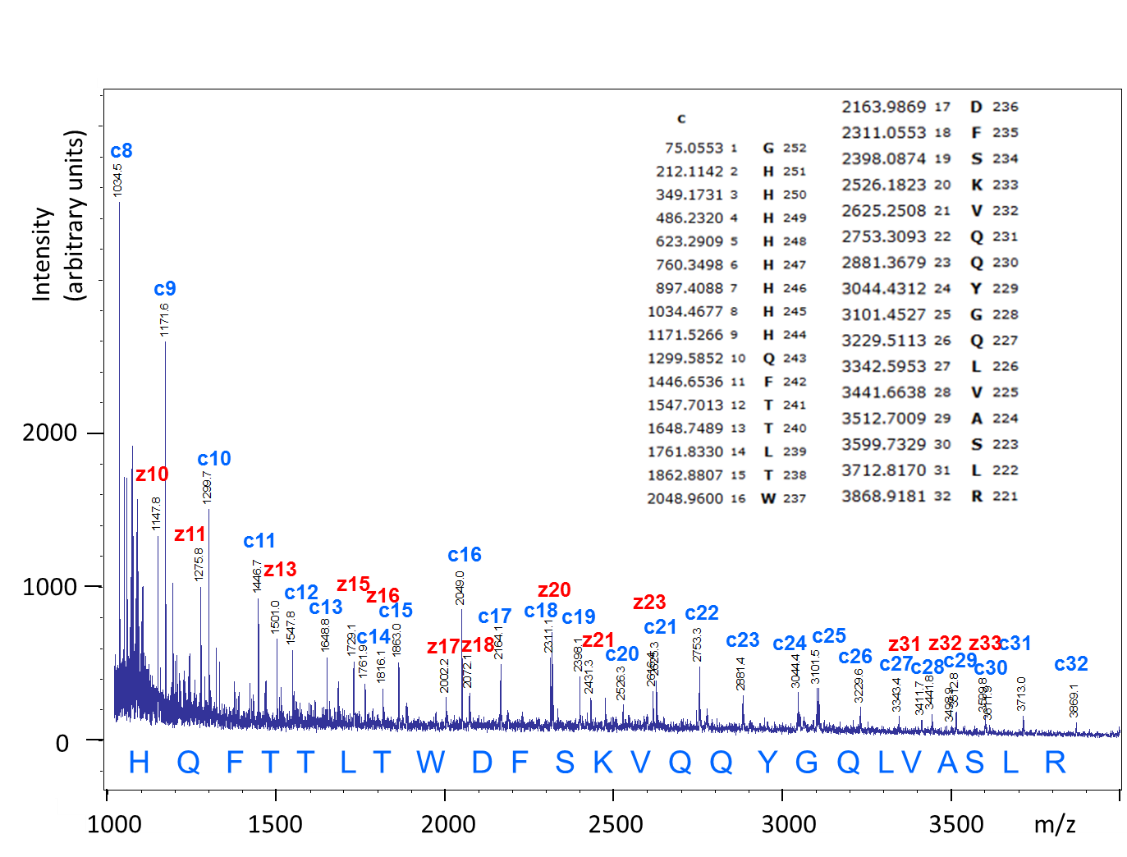


Fig. S2

MALDI ISD spectrum of purified recombinant agrostin on 1,5-DAN matrix; N-terminal ions are shown in blue, C-terminal ions in red; inset: Theoretical masses for N-terminal ions c1–c32 (without N-terminal Met).


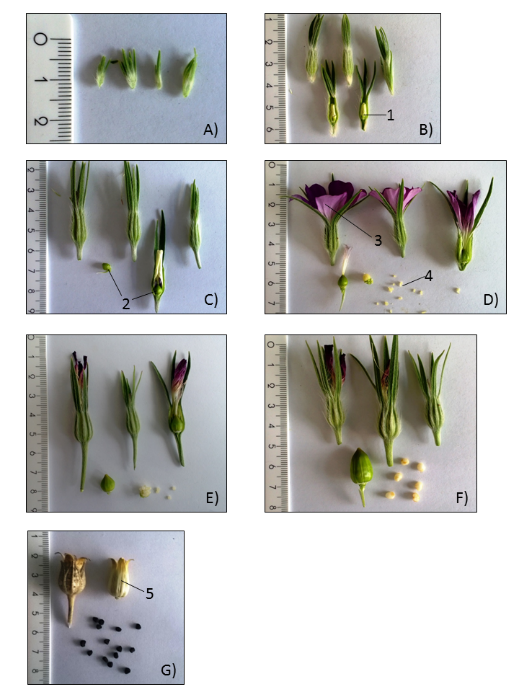

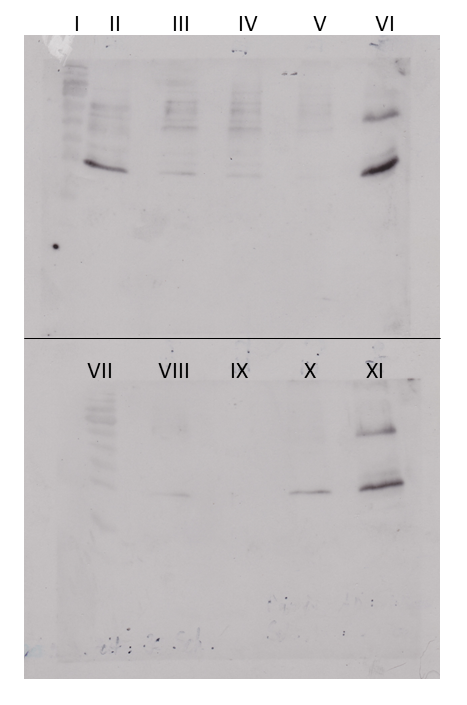


Fig.S3

Refers to fig. 3 in the main manuscript. Full images of (a) different development stages of *Agrostemma githago* L. and (b) western blot analysis of the extracts from stages a-g using the anti-agrostin antibody.
